# Identification of screw spacing on pediatric hip locking plate in proximal femoral osteotomy

**DOI:** 10.1007/s13246-023-01277-w

**Published:** 2023-05-22

**Authors:** Byeong Cheol Jeong, Tae Sik Goh, Chiseung Lee, Tae Young Ahn, Dongman Ryu

**Affiliations:** 1 Department of Biomedical Engineering, Graduate School, Pusan National University, Busan, 49241 Republic of Korea; 2 Biomedical Research Institute, Pusan National University Hospital, Busan, 49241 Republic of Korea; 3 Department of Orthopedic Surgery, School of Medicine, Pusan National University, Busan, 49241 Republic of Korea; 4 Department of Biomedical Engineering, School of Medicine, Pusan National University, Busan, 49241 Republic of Korea; 5 Medical Research Institute, Pusan National University, Busan, 49241 Republic of Korea

**Keywords:** Developmental dysplasia of the hip, Proximal femoral osteotomy, Pediatric hip locking plate, Screw spacing, Screw angle, Group pile

## Abstract

This study describes a computational analysis technique for evaluating the effect of screw spacing and angle on the pediatric hip locking plate system in proximal femoral osteotomy in pediatric patients having DDH with an aberrant femoral head and femoral angle. Under static compressive load conditions, the stresses of the screw and bone were examined as the screw spacing and angle changed. The spacing and angle of various screws were specifically considered as variables in this study based on the pile mechanism studied in civil engineering. As with the group pile mechanism, the tighter the screw spacing under static compressive loads, the more the overlapping effect between the bone stresses and the screws develops, increasing the risk of injuring the patient’s bone. Therefore, a series of simulations was performed to determine the optimal screw spacing and angles to minimize the overlapping effect of bone stress. In addition, a formula for determining the minimum screw spacing was proposed based on the computational simulation results. Finally, if the outcomes of this study are applied to pediatric patients with DDH in the pre-proximal femoral osteotomy stage, post-operative load-induced femur damage will be reduced.

## Introduction

Developmental dysplasia of the hip (DDH) encompasses all malformations associated with growing hips, including frank dislocation, subluxation and instability, and dysplasia of the femoral head and acetabulum [1]. The prevalence of these diseases are difficult to estimate owing to differences in the classification of the disease, the type of examination employed, and varying ability amongst clinicians [5, 6]. Furthermore, there are various strategies for treating DDH, and these approaches can be used in various ways, depending on the patient’s age. Infants up to 6 months of age who suffer from hip instability or dislocation are typically treated with a brace, such as a pavlik harness or abduction orthosis. Closed reduction and use of a hip spica cast can be used to treat patients, aged between 6 and 18 months, experiencing dislocation [7, 8]. Patients older than 12 to 18 months, as well as those who fail to achieve concentric hip reduction using closed procedures, are considered candidates for open surgical hip reduction [8]. Treatment approaches such as open surgical hip reduction with femoral shortening osteotomy or proximal femoral osteotomies can be used in patients older than 2 years [7, 8]. Proximal femoral osteotomy is an essential treatment option in children with hip problems [9, 10]. Proximal femoral osteotomy is used to treat DDH, Legg-Calve-Perthes disease, and slipped capital femoral epiphysis [9]. There exist fracture procedures employing various plates in proximal femoral osteotomy, but this study focuses on osteotomy using a pediatric hip locking plate. A pediatric hip locking plate provides primary stability by locking screws on the plate and femoral neck screw divergence. Furthermore, with proper angle adjustment, the pediatric hip locking plate ensures stable fixation and applies a significant fixing force even on osteoporotic femurs [14]. Following proximal femoral osteotomy with a pediatric hip locking plate, the lower body was usually immobilized with a spica cast. However, spica cast immobilization can cause several difficulties, which can be prevented by using foam as padding or a splint device [17].

Following these surgical procedures, it is routine to immobilize the lower body and determine surgical prognosis using computed tomography (CT). However, injuries to the femur around the screw caused by an external force can’t be quantified in this follow-up procedure. Furthermore, it is difficult to assess the risk of bone injury due to loading. Animal experiments or finite element analysis (FEA) can be used to detect and indirectly overcome these difficulties. For many years, in silico approaches based on FEA have been widely employed to examine disease mechanisms in other organs and have been widely used in femur-related research. From a biomechanical standpoint, Zhang et al. revealed the mechanical properties and theoretical foundation of a diaphyseal prosthesis with a tooth mechanism using an FEA approach [18]. Coquim et al.  evaluated the biomechanical performance of a metal plate and bone strut in correcting stubborn nonunions of mid-shaft segmental femur defects, which had not previously been systematically performed [19]. Takahashi et al. investigated the stability of a double-plate technique employing reversed contralateral locked compression-distal femoral plates for periprosthetic femoral fracture repairs while bearing full weight [20]. Samsami et al. proposed cannulated screws, dynamic hip screws with derotational screws, and proximal femoral locking plates as the most stable fixation for vertical femoral fractures [21].

Despite these FEA studies on the fixation of various fractures, research on proximal femoral osteotomies for patients with DDH has been limited. Furthermore, in a clinical investigation of proximal femoral osteotomies in children, the absolute values for plate and screw standards have not been quantitatively established.

Therefore, the finite element method was used in this study to investigate the variation of bone damage according to the screw specifications in pediatric hip locking plates with proximal femoral osteotomy. Bone damage assessments can assist in establishing the correct quantity, spacing, and angle of screws when setting up a treatment approach before surgery, which can aid in the post-operative management of young children. The spacing and angles of the screws addressed in this study were specifically introduced from the standpoint of civil engineering. In the case of screw spacing, various spacings have been investigated by applying the civil engineering principle of group piles to determine the ideal spacing [22, 23]. It is critical to maintain an optimum distance between piles during the installation stage, because zones of overlap occur in the stress field between the piles. Based on these principles, the stresses that occur in the femur due to screw spacing were investigated in this study. Furthermore, a series of simulations were performed based on the screw’s angle to improve the screw’s fixing force and reduce stress on the bone by expanding the contact area between the femur and the screw.

## Methods

### Finite element modeling

In this investigation, a CT image of a girl with advanced DDH (10 years old, mass of 17 kg, and height of 120 cm) was used to extract a 3D model of the femur to replicate the femur of a patient with advanced DDH. For the patient’s right femur, the angle between the femoral head and the femur was measured to be approximately 137.2°. The study was conducted in accordance with the declaration of Helsinki and approved by the Institutional Review Board of Pusan National University Hospital (IRB No. 2008-010-094). The patient voluntarily provided written informed consent. Written authorization of participants to publish data, including personal identifying information, was also collected.

**Fig. 1:**
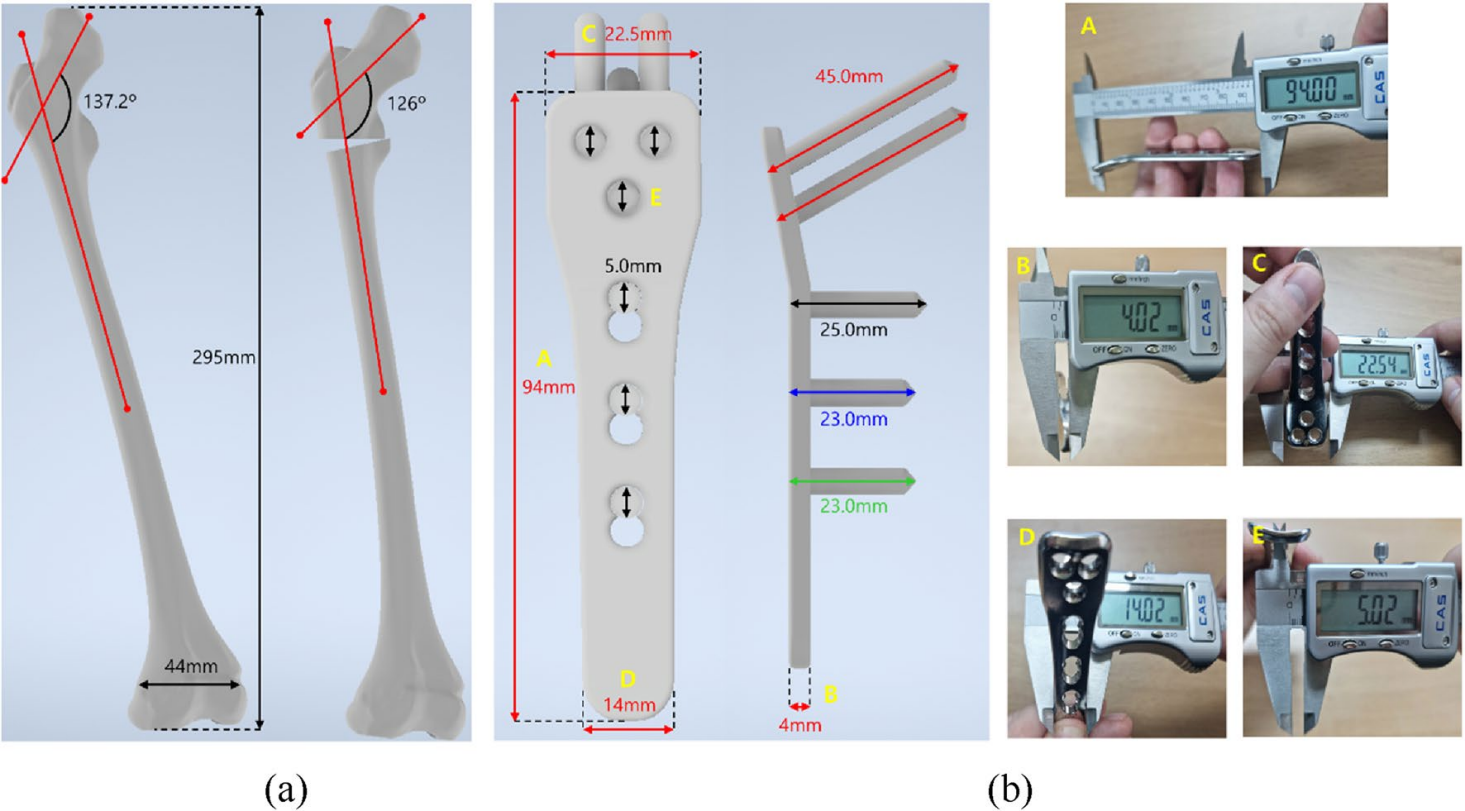
3D modeling and dimensions of **a** the femur, **b** the pediatric hip locking plate

Using 3D Slicer software, the previously obtained CT image data was transformed into a finite element model of the left femur. Osteotomy was used to cut and reshape the bone in this 3D virtual patient model, and the angle between the femoral head and femur was corrected to 126°, as shown in Fig. 1a [24].

In addition, the pediatric hip locking plate (LCP Pediatric Plate System, DePuy Synthes company, USA), used in this 3D virtual patient model, was measured using a digital caliper and converted to a finite element model, as shown in Fig. 1b. The screw holes in this pediatric hip locking plate were set to a diameter of 5.0 mm, and the location and number of screw holes were set to three in the direction of the femoral head and three in the direction of the femur. Based on the product specifications, the diameter and length of the screws were set to 5.0 mm and 23–45 mm, respectively. After selecting the positions of the screws inserted into the femoral head, the screws inserted in the direction of the femur were positioned based on the groove of the pediatric hip locking plate, and they were vertically fixed with the pediatric hip locking plate. Finally, ABAQUS (Version 2022, Dassault Systemes, Simulia, USA) finite element program was used to perform three-dimensional FEA, with a total of 1,496,941 solid elements in the finite element model. The femur and pediatric hip locking plate were composed of C3D4 element types in the form of tetrahedrons of sizes 0.2 and 0.5 mm, respectively, whereas the screw was composed of C3D8R element types in the form of hexahedrons of size 0.3 mm.

### Material properties, boundary conditions, and loading conditions

**Fig. 2:**
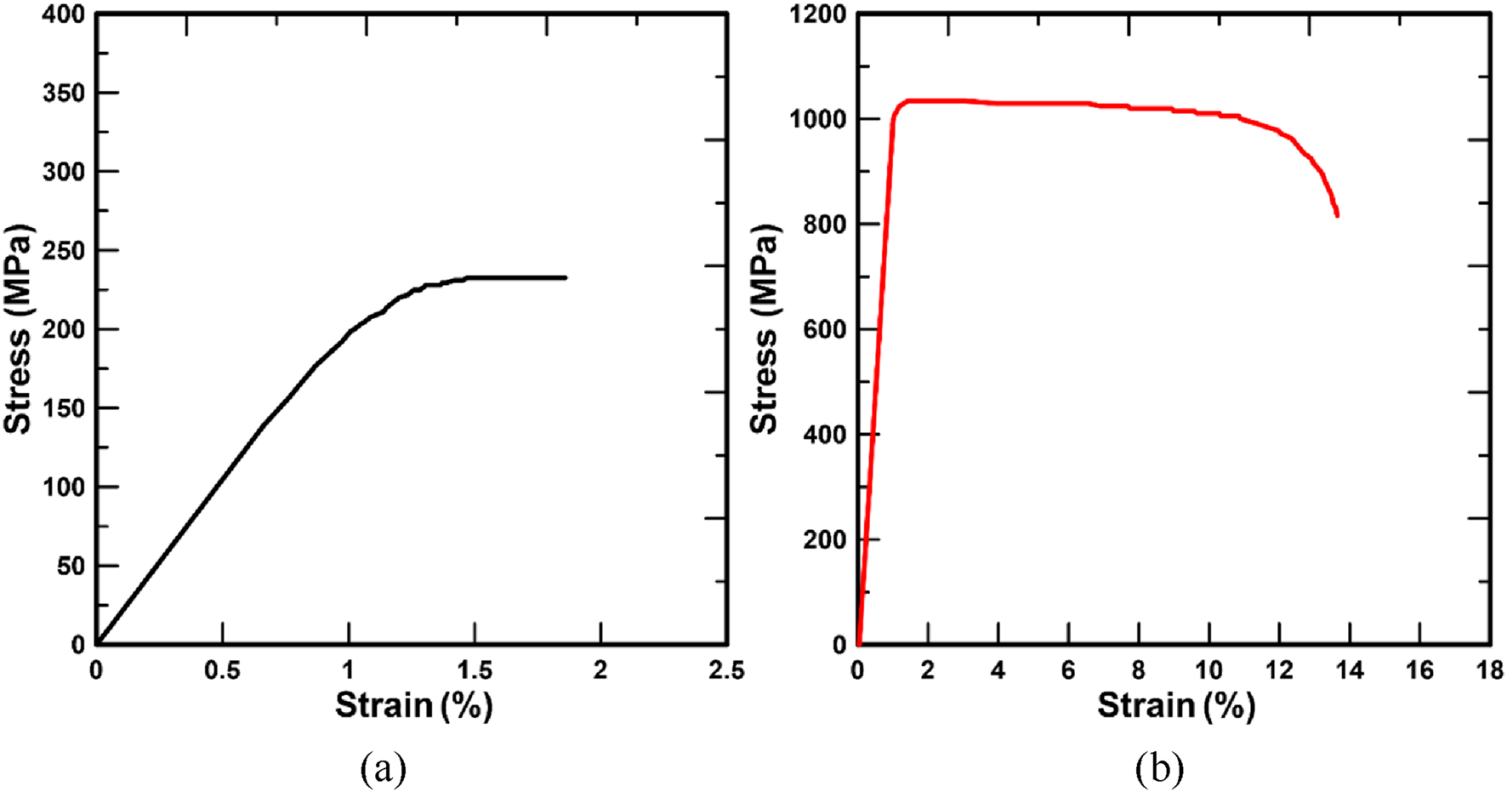
Stress–strain curves of **a** cortical bone and **b** titanium alloy (Ti6Al4V) [25, 26]

**Fig. 3:**
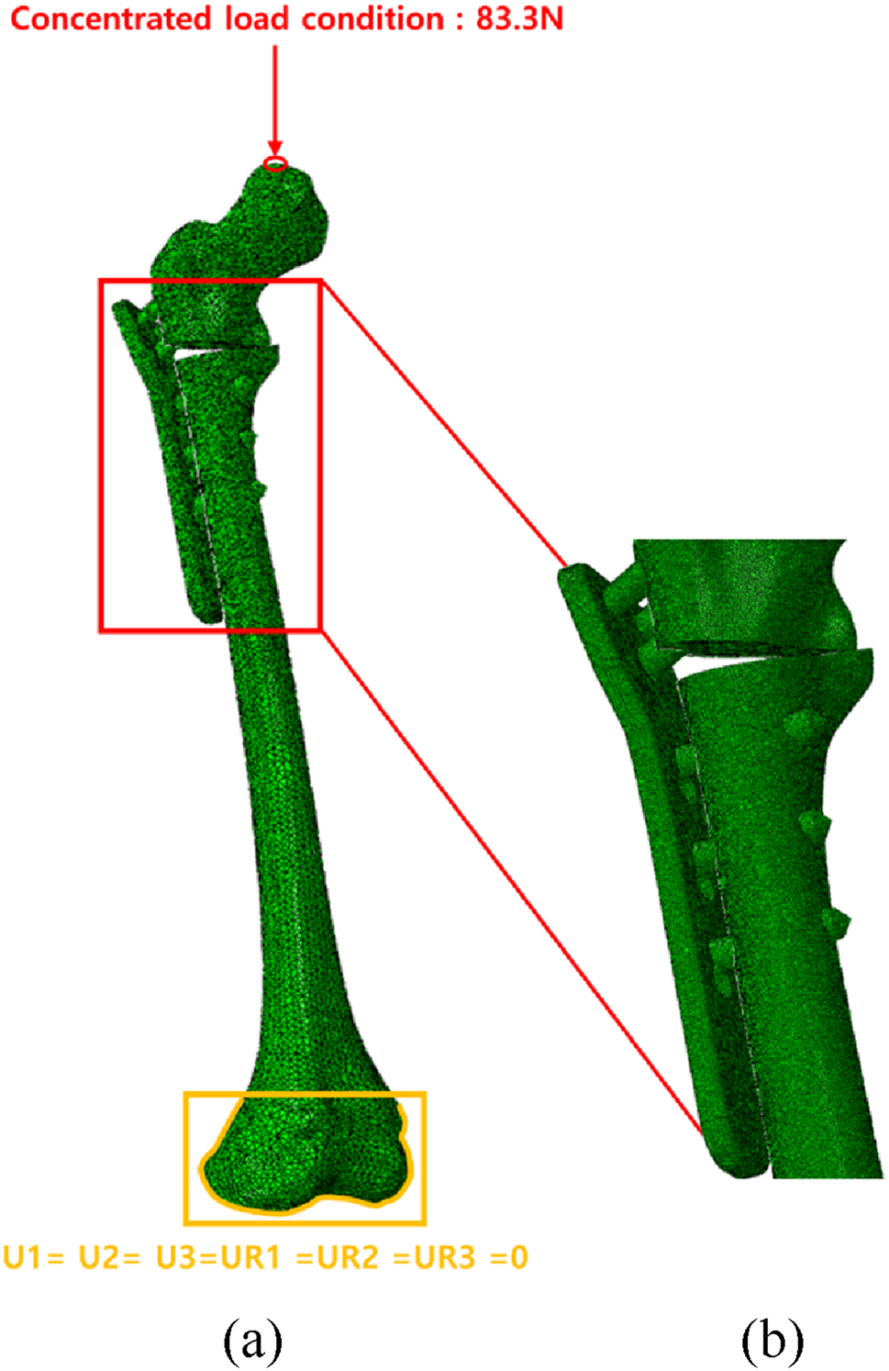
**a** The boundary and loading conditions for FEA, and **b** combination of the femur, pediatric hip locking plate, and screws

Table 1 shows the Young’s modulus, Poisson’s ratio, density of the femur, pediatric hip locking plate, and screw used in FEA. The stress–strain curve, obtained experimentally, was applied to FEA to precisely simulate the elastoplastic behavior of the cortical bone and titanium alloy, as shown in Fig. 2 [25, 26]. Figure 3 shows the boundary and loading conditions of the finite element model. Femur movement was restrained with boundary conditions by fixing the lowest part of the femur, as shown in Fig. 3a. The contact conditions between the screw and pediatric hip locking plate and between the screw and femur were designed as tie conditions to simulate the same behavior. Furthermore, this study focused on examining the behavior of the femur under stress, depending on the screw spacing and angle, and the screw and pediatric hip locking plate were generated as a finite element model, as shown in Fig. 3b. FEA was performed with the compressive load by establishing a concentrated loading condition at the top of the femoral head under the loading condition. As shown in Fig. 3a, the concentrated load was 83.3 N, under the assumption that half of the patient’s body weight was the upper body weight. In this study, the maximum load under static compressive loading conditions was determined to be half of the patient’s body weight.

**Table 1:** Material properties of cancellous bone, cortical bone, hip plate, and screw [25]

Materials	Density (ton/mm^3^)	Young’s modulus (MPa)	Poisson’s ratio
Cancellous bone [27, 29]	2.08e−009	137	0.3
Cortical bone [25, 27, 29]	1.60e−009	17,340	0.3
Pediatric hip locking plate & screw (Ti6Al4V) [26, 28]	4.429e−009	100,968	0.31

### FEA scenario

**Fig. 4:**
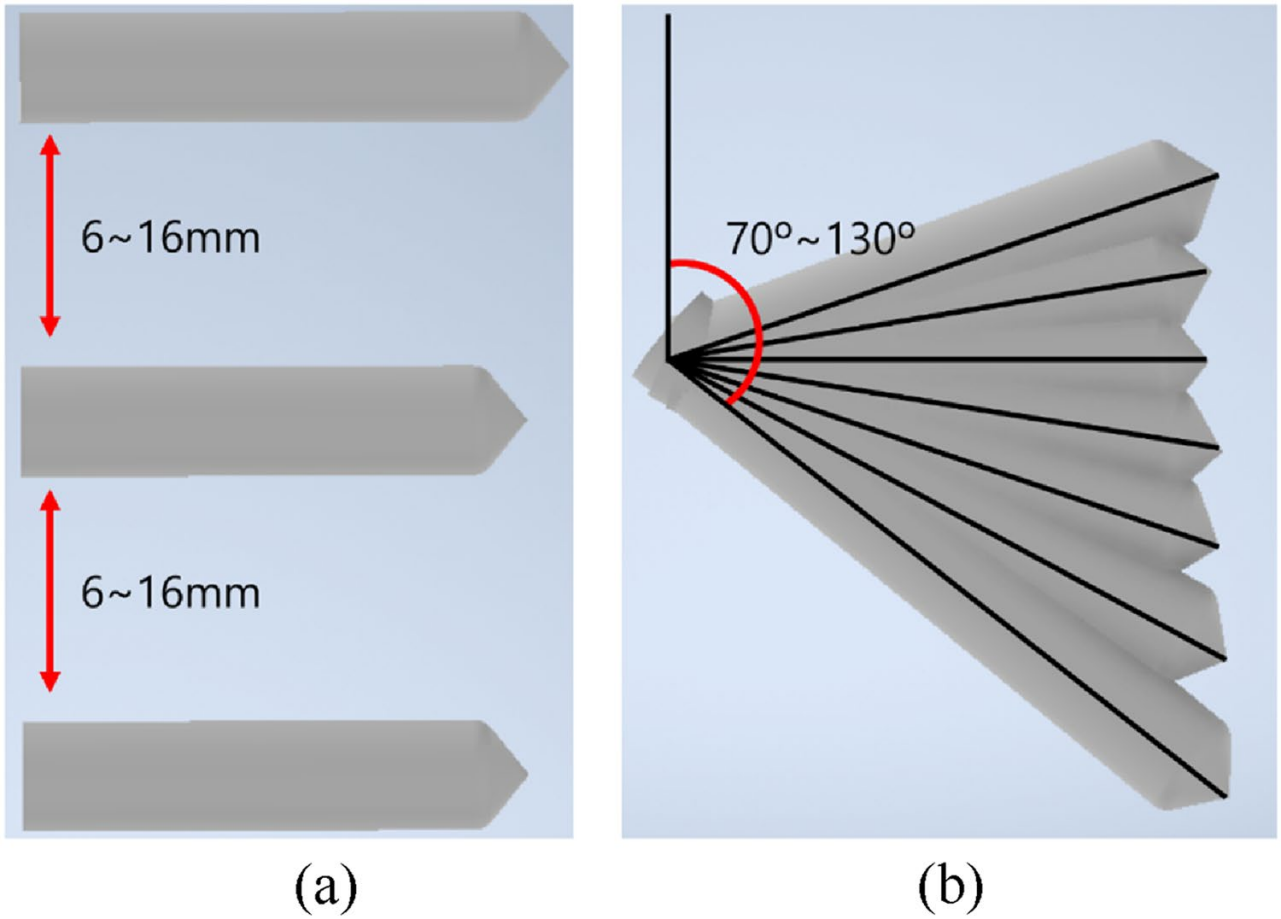
**a** The spacing and **b** the angle of screws in the FEA scenario

In this study, an FEA scenario was established with the spacing and angle of the screw to investigate the behavior of the bone under stress and relate it to the spacing and angle of the screw. The screw distance was considered to range from 6 to 16 mm (at intervals of 1 mm), whereas the angle was considered to range from 70° to 130° (at intervals of 10°). When modifying the distance between the screws attached to the femur, the highest of the three screws was held in place in all finite element models, whereas the other two screws were moved according to the scenario. Only the lowest of the three screws was moved when setting the angle. In addition, because of the screw-to-screw overlap, the case for 70° of the most inferior screw with screw spacings of 6 and 7 mm was removed from the scenario. Because screws attached to a single cortical bone of the femoral shaft have a weak fixation force, connecting screws to both cortical bones is recommended. In addition, during the process of securing the screw to the cortical bone, if the screw protrudes excessively from the cortical bone on the opposite side, it may damage the soft tissue beyond the cortical bone [30]. Therefore, the lowest of the three screws was fixed to both cortical bones with minimal protrusion, and the screw length increased as the distance between the cortical bones expanded owing to the modification of the screw angle. The assumption that as the screw spacing was reduced, the distribution of overlapping stress and a large amount of stress were generated in the bone was obtained from the mechanism of group piles installed in the ground. It was assumed that, as the area in contact with the femur expanded, the angle of the screw would contribute to an increase in the fixation force. As a result, the criteria for identifying the optimal screw spacing was addressed in this study, as well as the behavior of the bone under stress as a consequence of screw angle variation. Therefore, in this study, as indicated in Table 2; Fig. 4, a total of 75 series of simulations were performed with various screw spacings and angles. In addition, a screw-spacing formula based on the pile-spacing formula utilized in a group pile mechanism is proposed.

**Table 2:** FEA scenario for spacing and angles of screws

Variable	Value										
Distance between screws (mm)	6	7	8	9	10	11	12	13	14	15	16
Angle (°) [interval: 10]	80 – 130	80 – 130	70 – 130	70 – 130	70 – 130	70 – 130	70 – 130	70 – 130	70 – 130	70 – 130	70 – 130

## Results

### Stress of the screw in the static compressive loading condition

**Fig. 5:**
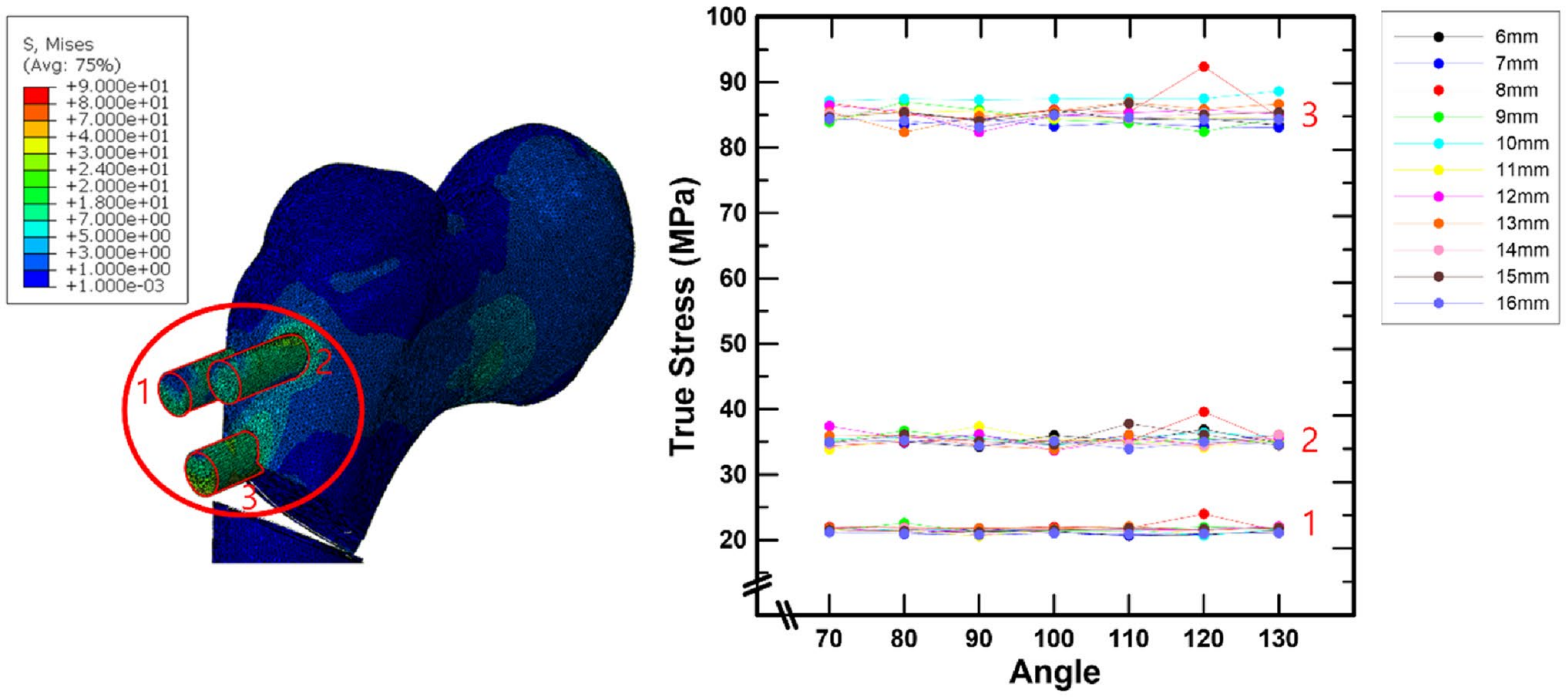
Screw stress in the femoral head as a consequence of screw spacing and angle

**Fig. 6:**
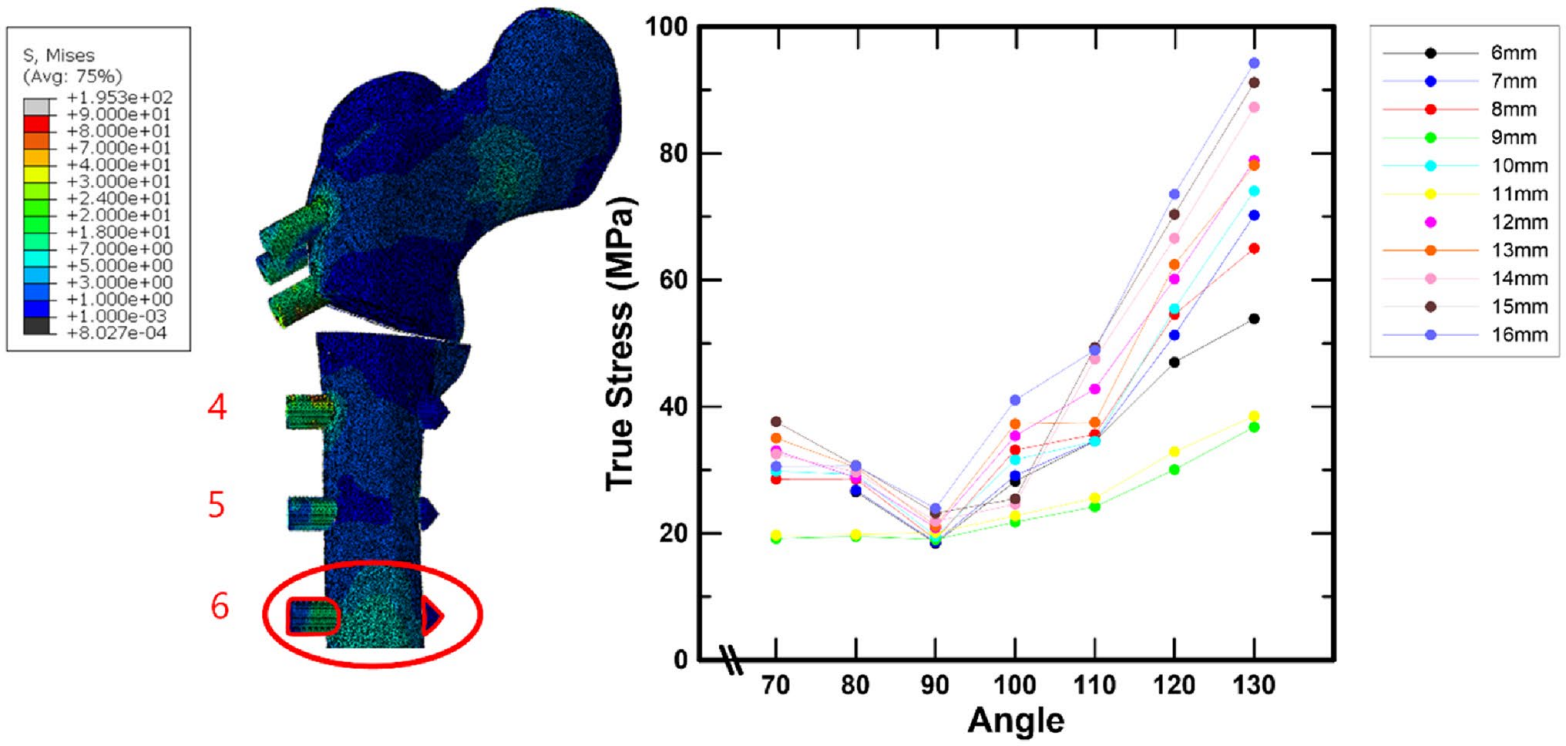
No. 6 screw stress at the femur based on spacing and angle

Under static compressive loading conditions, the series simulation results for the stress of the screw according to the spacing and angle of the screw are reported in this section for a patient who underwent proximal femoral osteotomy for DDH. The stress in a screw was investigated by separately distinguishing between screws inserted into the femoral head and screws inserted into the femur (Figs. 5 and 6). Figure 5 shows the variation in the maximum stress in the three screws placed into the femoral head based on the insertion methods of the other screws.

The variation in stress due to spacing and angle could not be demonstrated in the screws placed in the femoral head. However, the stress varied depending on the screw location. The average stresses for screws 1 and 2 in Fig. 5 were 21.48 MPa [± 0.8814 (standard deviation, SD)] and 35.33 MPa (± 0.9728), respectively, which were lower than the average stress for screw 3 at 85.13 MPa (± 1.5620).

Figure 6 shows the stress, as a consequence of the screw angle and spacing, in screw 6 placed in the femur. The variation in stress according to the angle and spacing could not be validated in the cases of screws 4 and 5 (Fig. 6). However, a difference in the angle and spacing was observed for screw 6.

### Stress of the bone in the static compressive loading condition

**Fig. 7:**
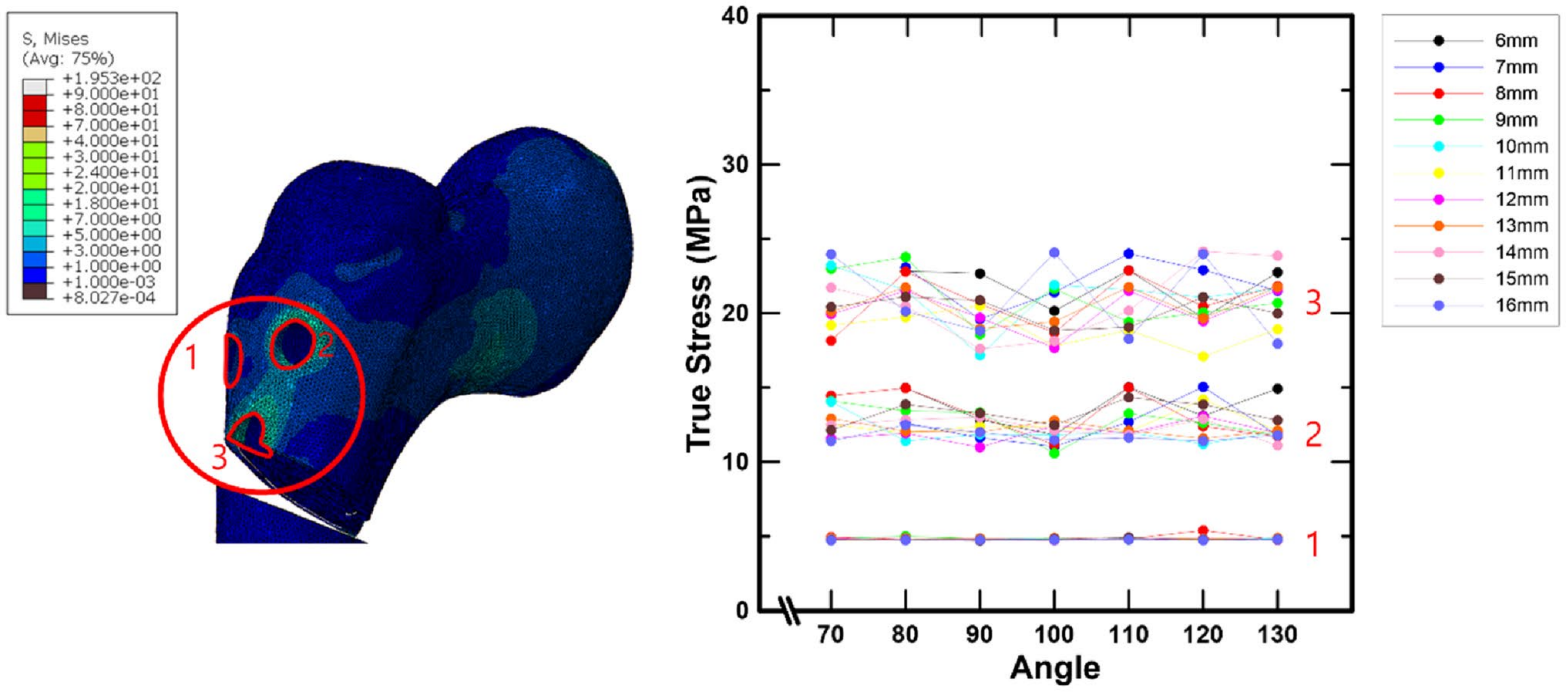
The bone stress around the screw placed into the femur head by the spacing and angle of the screw placed into the femur

The stress in the bone surrounding the screw inserted into the femoral head is depicted in Fig. 7. As with the previously measured stress on the screw, the greatest bone stress was observed at the location of screw 3, and it can be seen that there was a minor difference in the bone stresses depending on the screw position. Furthermore, the stresses in the bone around the screw inserted into the femoral head did not vary significantly as the angle and spacing of the screw inserted into the femur were changed.

**Fig. 8:**
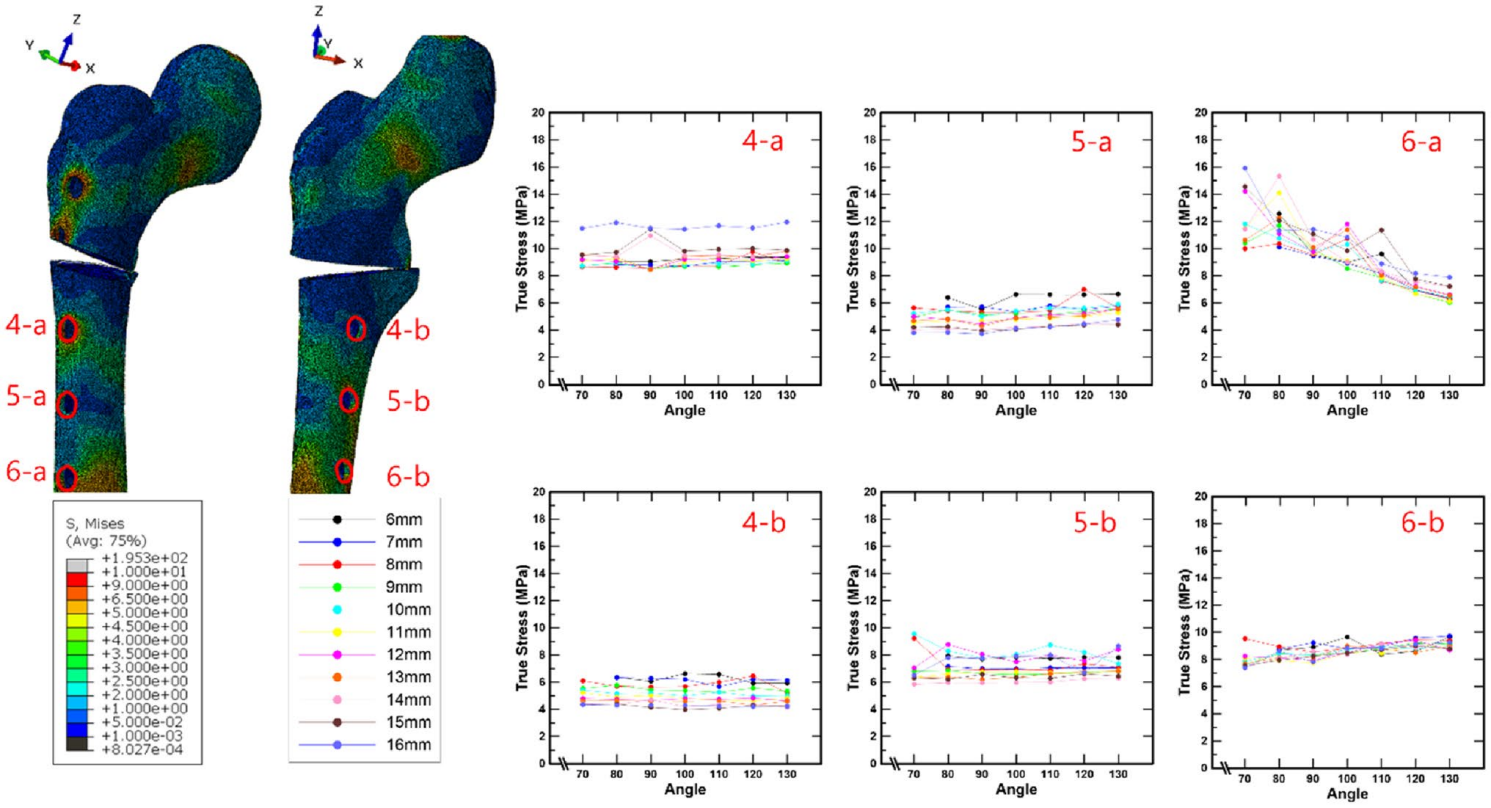
The bone stress around the screw placed into the femur in relation to the spacing and angle of the screw (**a** entry points of screws, **b** exit points of screws)

In the case of the bone stresses around the screw placed in the femur, it can be seen that the bone stresses around one screw have a different tendency than the bone stresses around the other screw, as shown in Fig. 8. In Fig. 8, positions 4-a, 5-a, and 6-a indicate the entry points of the screws attached to the femoral shaft, whereas positions 4-b, 5-b, and 6-b indicate the exit points. Moreover, the average stress in this section is the average value of all the bone stresses calculated according to the spacing and angle in a zone. At locations 4-a and 4-b, the average stresses were 9.42 MPa (± 0.8814) and 5.05 MPa (± 0.7247), respectively. Locations 5-a and 5-b had average stresses of 5.05 MPa (± 0.7426) and 7.11 MPa (± 0.8198), respectively. No clear trends related to screw spacing and angles have been observed. However, at location 6-a, the stress in the femur decreased as the angle of the six screws increased, whereas at location 6-b, the stress in the femur increased loosely as the angle of the six screws increased.

**Fig. 9:**
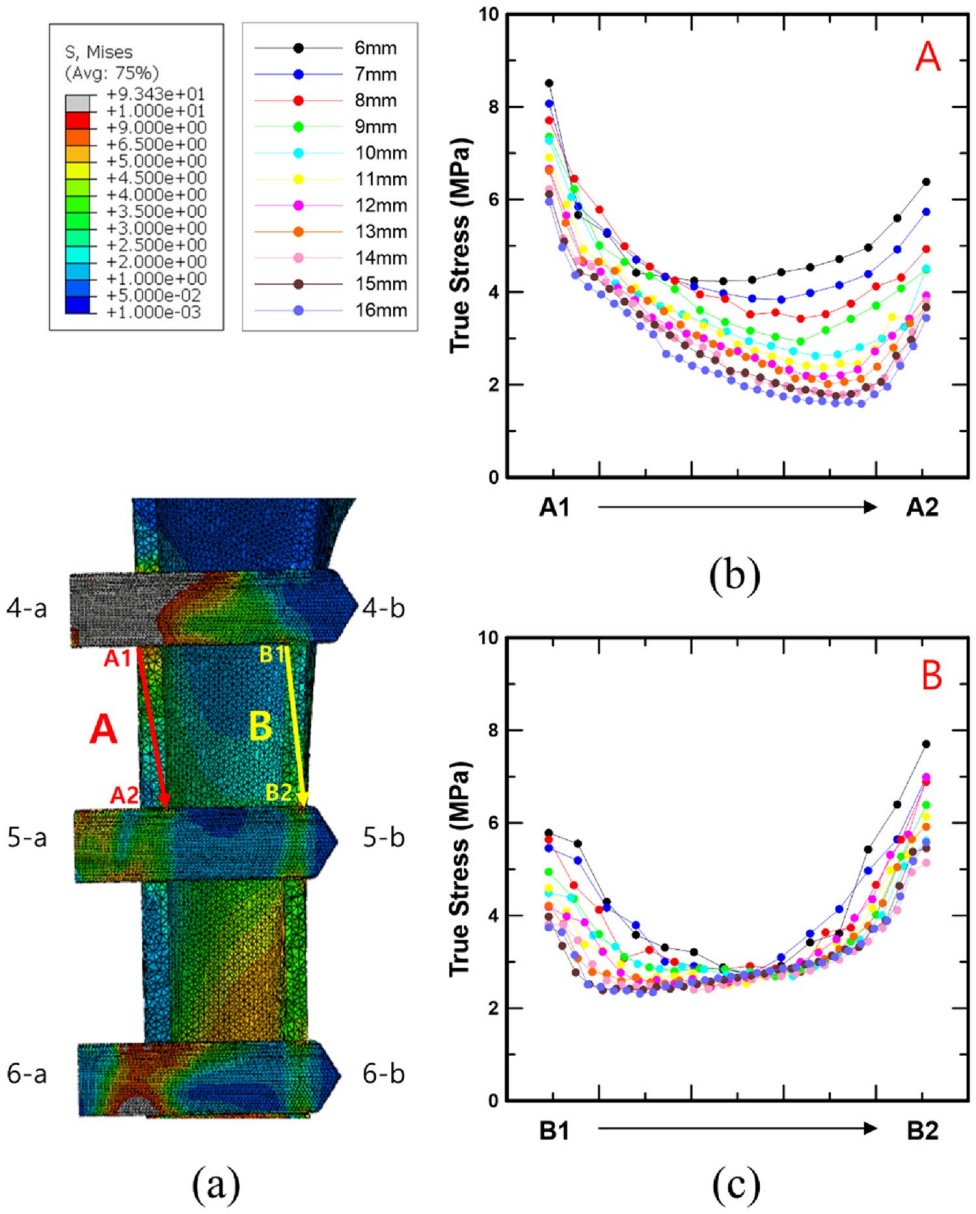
**a** The method for evaluating the stress distribution around the screw placed into the femur, as well as the results of cortical bone stress measurements on the **b** lateral and **c** medial sides of the femur between screws 4 and 5, based on the screw spacing and a 90° angle

Figure 9 shows the cortical bone stress distribution between screws 4 and 5 based on the screw spacing and a 90° screw angle. The stresses at the nodes which overlap the straight line linking the points of maximum stress at 4-a and 5-a, and the points of maximum stress at 4-b and 5-b in the femur cross-section were measured. As shown in Fig. 9b and c, the stresses in the femur decreased as the screw spacing increased for A (A1 to A2) and B (B1 to B2). In case A, the minimum bone stress decreased significantly as the screw spacing increased, as a result of assessing the minimum stress at each screw spacing and the variances between the minimum values (Table 3). It is clear that the decreasing rate of these minimal values decreased by 5% when the screw spacing was 11 mm or larger. However, in case B, it was established that the rate of decrease of the minimum values increased significantly when the screw spacing was 11 mm or larger.

**Table 3:** The bone stress minimum value for each screw spacing in Fig. 9 and the difference between the minimum values

Variable	Value										
Distance between screws (mm)	6	7	8	9	10	11	12	13	14	15	16
Minimum value of A in Fig. 9 (MPa)	4.24	3.84	3.42	2.93	2.63	2.38	2.18	2.01	1.78	1.76	1.59
Difference (%) [based on 6 mm]	–	− 9.5	− 19.2	− 30.8	− 38.0	− 43.8	−48.5	− 52.5	− 57.9	− 58.6	− 62.5
Minimum value of B in Fig. 9(MPa)	2.70	2.74	2.78	2.69	2.69	2.50	2.46	2.46	2.40	2.39	2.32
Difference (%) [based on 6 mm]	–	1.5	3.1	− 0.4	− 0.3	− 7.4	− 9.0	− 8.8	− 11.1	− 11.7	− 14.3

**Fig. 10:**
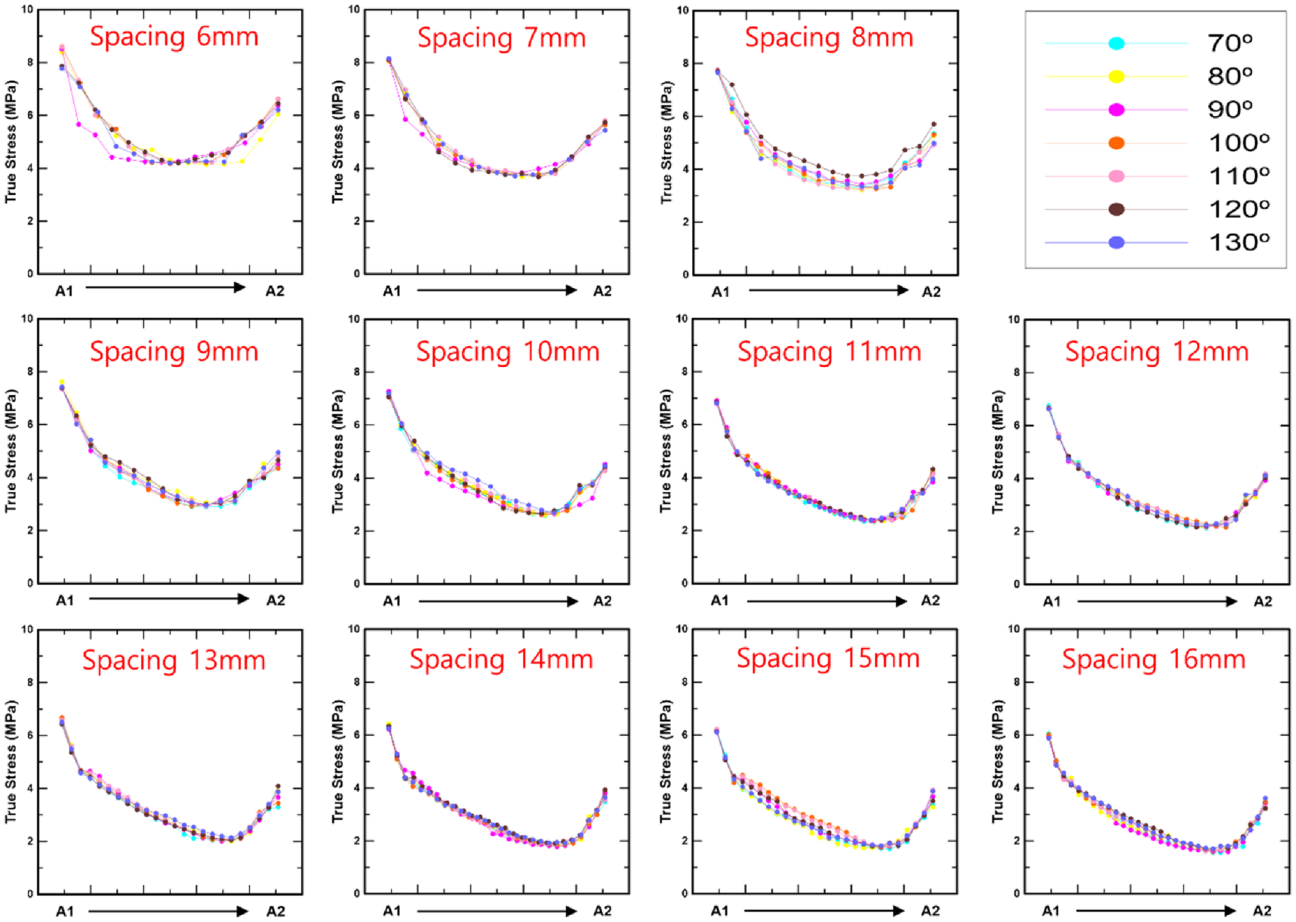
The bone stress distribution on the femur’s outside between screws 4 and 5 according to screw angle and spacing

**Fig. 11:**
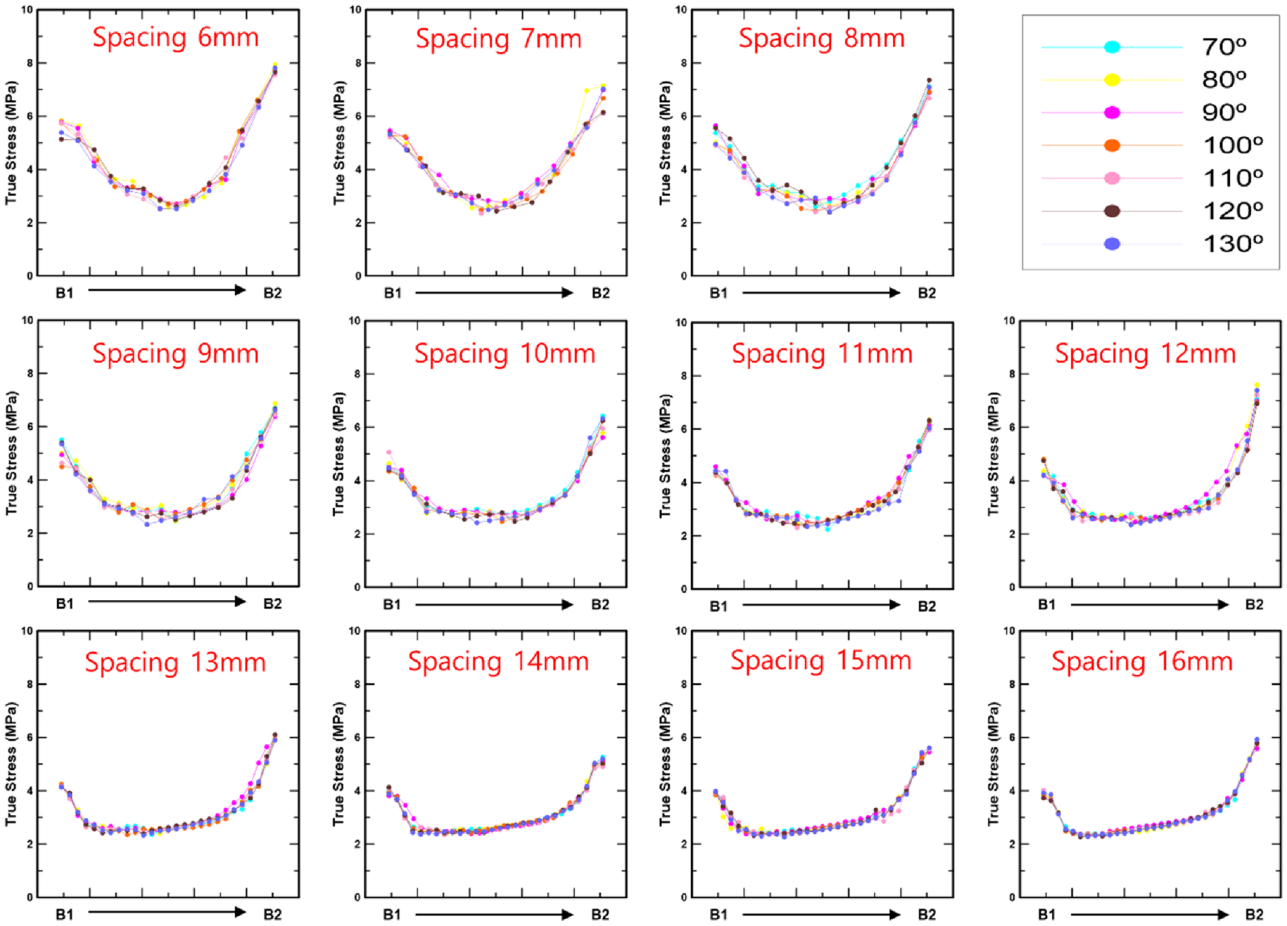
The bone stress distribution on the femur’s inside between screws 4 and 5 according to screw angle and spacing

Figures 10 and 11 show the bone stress distributions of A and B as a consequence of spacing and angle, respectively. It is difficult to identify a significant difference in the bone stress distribution according to the screw angle in the results for A and B. However, the variation in the bone stress distribution as a result of spacing may be clearly seen. In case A, the minimum bone stress distribution decreased from the upper left to the lower right as the screw spacing increased. In addition, in the case of B, the trend of the minimum bone stress is downward, going from the upper right to the lower left. Moreover, in Figs. 10 and 11, the X-axis is divided into 10 equal parts and expressed as a ratio, and the movement of the minimum value of the bone stress distribution in the femur may be quantitatively described as a result (Table 4). In case A, the minimum value appeared in a similar position at screw spacings ranging from 6 to 9 mm, and it is obvious that the minimum value was deflected to the right from screw spacings ranging from 10 mm and greater. The minimum value in the bone stress distribution was slanted to the left overall in case B, but the criterion for spacing could not be definitively validated.

**Table 4:** The movement in the position of the minimum value in the bone’s stress distribution as a result of screw angle and spacing based on the Figs. 10 and 11

Variable	Value										
Distance between screws (mm)	6	7	8	9	10	11	12	13	14	15	16
Minimum value of A in the Fig. 10 (MPa)	4.14	3.68	3.22	2.90	2.59	2.33	2.13	2.01	1.78	1.70	1.56
Position of the minimum value [X-axis: based on 1 to 10]	7.00	6.54	7.00	7.00	7.50	7.55	7.55	7.65	8.00	8.20	8.14
Minimum value of B in Fig. 11 (MPa)	2.50	2.34	2.39	2.33	2.43	2.25	2.34	2.33	2.36	2.27	2.27
Position of the minimum value [X-axis: based on 1 to 10]	5.15	4.86	5.80	4.60	4.71	5.71	4.68	4.46	3.33	3.89	2.55

## Discussion

In this study, a computational simulation was performed to quantitatively evaluate the effectiveness of the existing pediatric hip locking plate system used in proximal femoral osteotomy in children with DDH who had an unstable femoral head and femur angle. The spacing or angle of the screws placed into the femur can help distribute the load stresses in the bone. As a result, the ideal pediatric hip locking plate system was determined in this study by examining the screw and the bone stress around it induced by different screw spacings and angles. In addition, we present a formula for determining screw spacing to derive the ideal pediatric hip locking plate system for the environmental parameters of DDH examined in this study.

The insertion direction of the screw in the existing pediatric hip locking plate was separated into a screw placed into the femoral head and a screw inserted into the femur. In particular, it can be proven that there is no significant change in the stresses of a screw inserted into the femoral head owing to variations in the spacing and angle of the screws placed into the femur under a static compressive load. It should be emphasized that the function of the screw inserted into the femoral head is to strengthen the fixation force of the modified femoral head and pediatric hip locking plate to seat the patient’s femur in the optimal position. However, under static compressive load conditions, the stress distribution of the screws placed in the femoral head differed according to their insertion position. In the case of screws 1 and 2 in Fig. 5, the average stresses were 22 MPa and 35 MPa, respectively, which are much lower than the average stress of screw 3 placed at the bottom of the femoral head (85 MPa). This is because screw 3, which is placed into the femoral head, is closest to the bone incision position, which has a significant impact on plate resistance and static compressive load.

Furthermore, screws 4 and 5 placed into the femur did not result in significant outcomes in terms of screw spacing and angle. However, in the case of screw 6, which was placed in the femur, the screw stress increased as the angle increased. When the screw spacing was 9 and 11 mm, the variation in screw stress according to the angle increased only slightly. This is due to the fact that each position differs slightly in the process of determining the maximum stress from the screw. Moreover, a series of simulations for screw spacing indicated that the stress on screw 6 increased as the screw angle increased from 90° to 130°, with a maximum stress value of 94.27 MPa. Considering the material properties of the screw, it can be confirmed that the screw of the pediatric hip locking plate is not greatly influenced by the screw angle because a stress of 100 MPa or less is considered to be a very low value when compared to the yield stress.

The bone stress around the screw placed into the femoral head did not alter significantly depending on the screw spacing and angle, although there was a modest variance depending on the screw location. In particular, the stress on screw 3 was significantly different compared to other screws in the stress analysis, whereas the bone stresses around screw 3 were overall higher than the stresses in other locations in the stress analysis. However, it can be seen that the difference in bone stresses around the screw inserted into the femoral head was not large. This indicates that the static compressive load has a greater effect on the screw than on the bone in the femoral head region.

In addition, the results of the bone stress analysis demonstrated that only the bone stress surrounding screw 6 changed according to the screw angle. This means that as the screw angle increases, so does the contact area between the bone and the screw, reducing the stress that can occur in the bone owing to the static compressive load. Based on these results, it is hypothesized that, by reviewing the thickness of the lateral and medial femur in the CT-analysis stage of patients with DDH prior to surgery, the screw angle can be altered to reduce stress on the femur. For example, if the lateral cortical bone of the femur is thinner than the medial cortical bone, the angle is increased to reduce load damage because the lateral cortical bone is more susceptible to stress. Furthermore, to quantify the stress distribution generated in the bone around screws 4 and 5 inserted into the femur, this study was divided into sections A (A1 to A2) and B (B1 to B2), as shown in Fig. 9, and the stresses of the nodes on the corresponding straight line were investigated. As a result, as the screw spacing increased, the minimum value of bone stress in section A shifted from the upper left to the lower right. The bone stress dropped rapidly as the screw spacing increased from 6 to 10 mm, and the width of the decline in bone stress decreased dramatically at 11 mm or greater. This implies that there is a similarity to the effect of group piles used in civil engineering. In the case of group piles, the effect of overlapping stress occurs dependent on the spacing of the piles. Thus, the narrower the screw spacing, the higher is the stress generated in the bone owing to the overlapping effect of the stress [31–33].

In proximal femoral osteotomy, it is important to comprehend how the stress on the screw increases and the stress on the bone decreases according to the angle or spacing of the screw. Stresses due to external forces, such as compressive and tensile loads, are typically localized in the screws of DDH patients who have undergone proximal femoral osteotomy [34]. This phenomenon is believed to minimize bone damage by concentrating stress on screws with greater yield strength or ultimate strength than bone. Particularly, X-ray imaging is used to confirm that the bones and screws have successfully fused following surgery, and the patient’s status is examined by having them stand up with crutches. The condition of the patient’s bone at this time is predicted to be the most vulnerable state prior to the patient’s movement; therefore, in this study, the stress of the screw and bone was identified based on the angle or spacing of the screw. On this basis, the environmental conditions that minimize bone damage within the allowable stress range of the screw were derived. Therefore, if proximal femoral osteotomy is performed in patients with DDH while considering screw spacing, bone stress that may occur as a result of the patient’s behavior after surgery can be reduced, and the patient’s quality of life can be enhanced.

The piles should be constructed such that the force exerted by one pile on another is as small as possible [35]. Because the group pile evaluates the pile foundation spacing and skin friction to establish the pile foundation design, efficiency, and capacity, the spacing rules and calculation formulae are implemented throughout the pile foundation construction. Therefore, in this study, the aforementioned screw spacing variables were inspired by the mechanism of group piles used in civil engineering [35–37]. The efficiency of the piles varies depending on the pile spacing. According to the outcomes of studies conducted by Ilyas et al. and Wael et al., the efficiency of group piles decreases as the spacing between piles increases to three, four or more diameter lengths [35, 37]. Based on the outcomes of a study on group piles, the Korea National Railway recommended a method for determining the minimum center spacing of piles via pile foundation design guidelines [38].

For single piles,

1 $$\text{S}>1.5\sqrt{r\times L}.$$

For group piles,

2 $$\text{S}<1.5\sqrt{r\times L},$$

where S is the pile center-to-center spacing, r is the radius of the pile, and L is the length of the pile. Based on Eqs. (1) and (2), the existing calculation formula was modified in this study to propose the spacing of screws placed into the femur as follows:

3 $$\text{S}<1.5\sqrt{r\times L\times n},$$

where n is the number of screws. This revised proposed formula was modified based on the results of a series of simulations in which the superposition effect of the stress generated in the bone decreased with a screw spacing of 11 mm or greater.

However, this study has a number of limitations. First, when walking, the joint reaction force is typically calculated to be between 3.5 and 5 times the body weight [39]. In addition, the joint reaction force in a single-leg stance is commonly estimated to be 2.7 times the body weight [40] and is occasionally calculated to be up to six times the maximum body weight [39]. However, the loading condition considered in this study is half the subject’s body weight. In this study, we examined the loading condition that occurs when both feet first step on the floor using crutches in the patient’s recovery phase after proximal femoral osteotomy, where the majority of the load is believed to be distributed between the crutches and the normal leg. In particular, in the study by Seker et al., the joint reaction force in the double-leg stance was set at one-third of the subject’s body weight [41], whereas in the present study, the load condition was set at half of the subject’s body weight based on the worst-case scenario.

Second, the verification step of comparing the FEA results with the experimental results based on proximal femoral osteotomy was not performed in this study. Therefore, in this study, FEA was conducted considering the material properties of the actual bone to ensure the validity of the FEA results by referencing previous research [42–45].﻿ Furthermore, the actual material properties based on the stress–strain curves of the femur, plate, and screw were considered in this study, and simulations were conducted that could consider the plastic region as well as the elastic region of the material [25–29].﻿

Third, in this study, the femoral-bone properties of adults, not children, were considered, as were the femoral bone properties of healthy individuals. Obtaining femur samples from actual children and femurs from DDH patients presents numerous challenges. Therefore, in this series of simulations, we focused on determining the optimal surgical method based on the trend of the screw spacing and angle values.As per journal instruction funding is mandatory hence we processed acknowledgment statement as funding statement. Kindly check.Yes, I checked, and the funding statement is fine.

Fourth, this model only considered static compression, and other loading conditions may produce different results. In addition, because only one osteotomy technique was evaluated, different outcomes may be observed when similar load conditions are applied to other osteotomy techniques.

To overcome these problems, we plan to conduct additional study on cyclic and dynamic loading, the development of an experimental system capable of motion analysis, and various material characteristics that can consider deformed and children’s bones. This study can be considered the first step in conducting these future studies. Particularly, the findings of this study can be applied to the design phase of the pediatric hip locking plate to create an improved plate with the capacity to control screw spacing. In addition, we believe that the fixation-force effects of each screw angle, as determined in the preceding analysis, and the results of future studies can be utilized to improve upon the proximal femoral osteotomy methodology. Therefore, the findings of this study are expected to provide surgeons with guidelines for improved proximal femoral osteotomy and facilitate improvements in other surgical techniques.
